# Parasitic polymorphism of *Coccidioides* spp

**DOI:** 10.1186/1471-2334-14-213

**Published:** 2014-04-21

**Authors:** Bertha Muñoz-Hernández, Gabriel Palma-Cortés, Carlos Cabello-Gutiérrez, María Angeles Martínez-Rivera

**Affiliations:** 1 Laboratorio de Micología Médica, Depto. de Investigación en Virología y Micología, Instituto Nacional de Enfermedades Respiratorias Ismael Cosío Villegas, Calzada de Tlalpan No. 4502, Colonia Sección XVI Del. Tlalpan, 14080, México, DF, México; 2 Laboratorio de Micología Médica, Depto. de Microbiología. Escuela Nacional de Ciencias Biológicas (ENCB), Instituto Politécnico Nacional (IPN). Carpio y Plan de Ayala s/n, Colonia Casco de Santo Tomás, Del. Miguel Hidalgo, México, DF 11340, México

**Keywords:** Coccidioidomycosis, Diagnosis of *Coccidioides*, Parasitic mycelia forms of *Coccidioides*, Parasitic polymorphism of *Coccidioides*

## Abstract

**Background:**

*Coccidioides* spp. is the ethiological agent of coccidioidomycosis, an infection that can be fatal. Its diagnosis is complicated, due to that it shares clinical and histopathological characteristics with other pulmonary mycoses. *Coccidioides* spp. is a dimorphic fungus and, in its saprobic phase, grows as a mycelium, forming a large amount of arthroconidia. In susceptible persons, arthroconidia induce dimorphic changes into spherules/endospores, a typical parasitic form of *Coccidioides* spp. In addition, the diversity of mycelial parasitic forms has been observed in clinical specimens; they are scarcely known and produce errors in diagnosis.

**Methods:**

We presented a retrospective study of images from specimens of smears with 15% potassium hydroxide, cytology, and tissue biopsies of a histopathologic collection from patients with coccidioidomycosis seen at a tertiary-care hospital in Mexico City.

**Results:**

The parasitic polymorphism of *Coccidioides* spp. observed in the clinical specimens was as follows: i) spherules/endospores in different maturation stages; ii) pleomorphic cells (septate hyphae, hyphae composed of ovoid and spherical cells, and arthroconidia), and iii) fungal ball formation (mycelia with septate hyphae and arthroconidia).

**Conclusions:**

The parasitic polymorphism of *Coccidioides* spp. includes the following: spherules/endospores, arthroconidia, and different forms of mycelia. This knowledge is important for the accurate diagnosis of coccidioidomycosis. In earlier studies, we proposed the integration of this diversity of forms in the *Coccidioides* spp. parasitic cycle. The microhabitat surrounding the fungus into the host would favor the parasitic polymorphism of this fungus, and this environment may assist in the evolution toward parasitism of *Coccidioides* spp.

## Background

*Coccidioides* spp. is a dimorphic fungus and, in its saprobic or vegetative phase, it grows as a mycelium, forming a large amount of arthroconidia. In susceptible persons, arthroconidia induce dimorphic changes into spherules with endospores a typical parasitic form of *Coccidioides* spp. Additionally, the parasitic morphological diversity of mycelial forms has been observed by several authors; however, these fungal structures are unknown as parasitic forms and knowledge is required to make an accurate diagnosis [1-9].

In early research, our working group published a study, which showed 44 patients (all of them born in Mexico) who presented spherules (typical forms) and/or parasitic mycelial forms (atypical forms). In that work we presented socio-economics, co-morbidities, and clinical data, in addition to microbiological and radiological studies, finding an operational definition that included all of the cases: "Patients with pulmonary coccidioidomycosis with evolution of 2.5 years to 8 months (chronic), which included cough, hemoptysis, radiographic evidence of cavitary lesion and type 2 diabetes mellitus (co-morbidity) developed parasitic mycelial forms of the fungus", and we proposed the integration of this diversity of forms into the parasitic cycle of *Coccidioides* spp. [7,8].

Although parasitic mycelial forms of *Coccidioides* spp. have been found in ≥50% of patients with cavitary and chronic pulmonary coccidioidomycosis by our working group, these are not able to be diagnosed by themselves; thus, it is advisable to conduct a search of the spherules, when only cytological and histopathological methods for diagnosis are used and support this diagnosis by fungus culture or immunological test. The "atypical" hyphal forms of *Coccidioides* spp. can be confused with other fungal infections such as aspergillosis, hyalohyphomycosis, etc., and rounded fungal arthroconidia can be confused with blastoconidia, parasitic forms of *Blastomyses dermatititdis*, the ethiological agent causative of blastomycosis; therefore, we recommend confirmation of the diagnosis by searching for the typical parasitic structures of *Coccidioides* spp. spherules/endospores of this infection. However, if they are not observed in specimens, it is necessary to support the diagnosis with clinical and epidemiological data, with serological studies, or by isolation of the fungus. *Coccidioides* spp. has never been having as normal biota of humans, only isolates it in infectious processes. Knowledge of these "atypical" parasitic structures allows possible association with coccidioidal infection (an infection with a possible fatal outcome) and not to discard it, as occurs at present. This research is based on an earlier study in which typical and atypical parasitic fungal structures of *Coccidioides* spp were observed and we sought for greater diversity of parasitic fungus structures. Here we present a retrospective study of images of specimens of smears with 15% potassium hydroxide, cytology, and tissue biopsies of cytological and/or histopathological collections from patients with cavitary and chronic pulmonary coccidioidomycosis seen at the Instituto Nacional de Enfermedades Respiratorias (INER) in Mexico City, a tertiary-care institution. The diversity of the parasitic forms of *Coccidioides* spp. observed in clinical specimens were the following: i) spherules/endospores at different maturation stages; ii) pleomorphic cells, septate and branched hyphae, hyphae composed of ovoid, rectangular, and spherical cells); iii) arthroconidia (spherical, triangular, ovoid, or barrel-shaped); and iv) fungal ball formation (mycelium with septate hyphae and arthroconidia), Thus, the parasite polymorphism of *Coccidioides* spp. includes this morphological diversity.

In the present study, we emphasize our effort to present a greater diversity of parasitic forms of *Coccidioides* spp. observed in clinical specimens from patients with chronic cavitary pulmonary coccidioidomycosis (including specimens from patients previously studied, analyzed in greater detail), and we included a greater diversity of parasitic forms than those found in the earlier study. The knowledge of all these structures favors the performance of the accurate diagnosis of this infection.

## Methods

The present work is a retrospective study on patients with respiratory apparatus pathologies suggesting pulmonary coccidioidomycosis infection, who were referred to the Mexico City-based National Institute of Respiratory Diseases (INER) from 1992–2011, who were included in the study; all of these patients were born in Mexico. The current investigation has been performed with the approval of the INER Science and Bioethics Research Committee. The present research is based on an earlier study in which parasitic fungal structures, including typical and atypical *Cocciddioides* spp., were observed [8]. Inclusion criteria included medical records from patients with clinical, microbiological, and histopathological tissues slices and/or fixed smears, and fungus growth-confirmed diagnosis of coccidioidomycosis. Exclusion criteria included medical records from patients with non-confirmed microbiological diagnosis of coccidioidomycosis or records lacking information.

Laboratory and microbiological diagnosis included the following: i) direct smear with 15% potassium hydroxide (KOH); ii) isolates of *Coccidioides* spp. on Sabouraud agar and mycobiotic agar at 28°C until mycelium growth, *Coccidioides* identification was performed by colony and microscopic morphology, additionally, specific *Coccidioides*´ exoantigens were obtained from each isolate by the Standard & Kaufman method [10], the isolates were identified by Ouchterlony double immunodiffusion with specific antibodies (all of the isolates presented identity bands for *Coccidioides* spp.); iii) cytological and histopathological tissue slices; iv) serological tests performed to identify anti-*Coccidioides* spp. that included gel immunodiffusion, capillary precipitation, and Enzyme-linked immunosorbent assay (ELISA), and v) cellular immune response also evaluated with a coccidioidin sensitivity test.

Studies were performed based on Histopathological Collection tissue slices and on fixed smear samples of previous specimens from patients with coccidioidomycosis. Microscopic examinations were carried out in sputum and bronchial lavage or by brushing specimens, secretions from fistulae, lung tissue, lymph node, or skin tissue slices. Direct examination with 15% KOH was performed and Calcofluor white, fluorescent whitening cytology (CFW). Images included cytology and histopathology using Hematoxylin and eosin (H&-E), Gomori methenamine silver (GMS), Periodic acid Schiff (PAS), and Papanicolaou smear stains.

## Results

**Figure 1:**
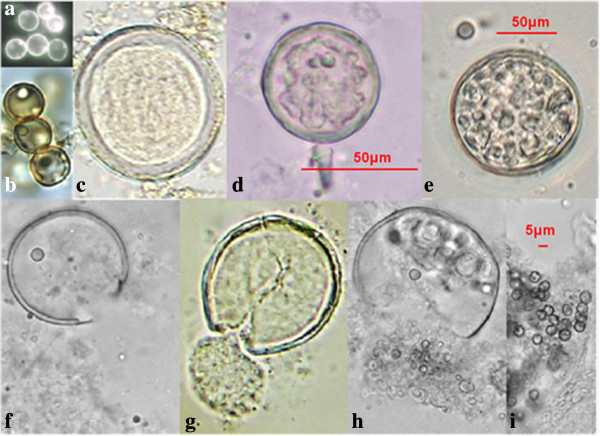
**Differentiation of spores into spherules. (a, b)** Differentiating spherical spores; **(c, d)** young spherules without endospores; **(e)** mature spherule with endospores; **(f, g, h)** expulsion of endospores, and **(i)** endospores free. **(a, b)** bronchial lavage and **(c, d, e, f, g, h, i)** sputum. **(a)** 40X, calcofluor white staining, and **(b)** 40X; **(c, d, e, f, g, h, i)** 100X, fresh examination with 15% KOH.

**Figure 2:**
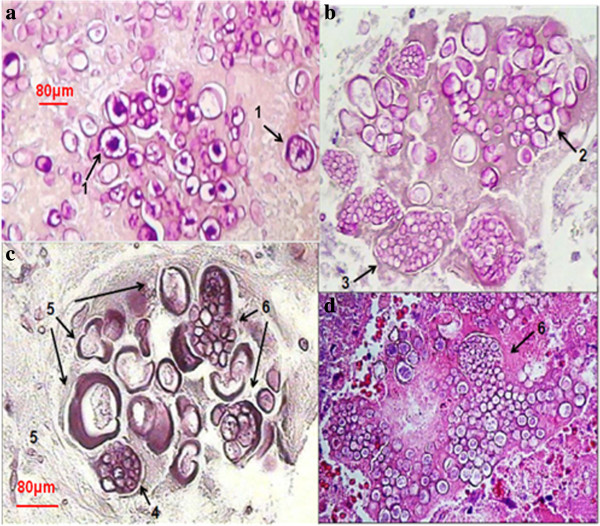
**Spherule differentiation in lung tissue. (a)** Segmentation and differentiation of endospores-1; **(b)** endospores free-2, morulares forms-3; **(c)** spherules with endospores-4 and without endospores-5; **(d)** spherules expelling endospores-6. 40X, staining with Periodic acid-Schiff (PAS). Arrows indicate the structures described.

The parasitic polymorphism of *Coccidioides* spp. is presented on fresh examination with 15% KOH, Calcofluor white (CFW) staining cytology, and histopathological images. These included the following: differentiation of spores and spherules in different steps of maturation, empty coat spherules, young differentiated spherules, mature spherules with endospores, spherule rupture expelling endospores (Figure 1). Phenotypic spherule variation, in (Figures 1c, 1d and 1e) can be confused with parasites such as *Emmonsia parva* (Coccidia and Adiaconidios) in its parasitic phase, due mainly to its thick cell wall. Spherical and ovoid shape of endospores expelled from *Coccidioides* (Figures 1h and 1i) must be differentiated from young blastoconidia of *Blastomyces* spp. or *Paracoccidioides* spp. In these images (Figure 2), one way observe different states of spherule maturation in lung tissue; from vacuole segmentation and differentiation into endospores (Figure 2a), spherule mature, cell wall degradation and endospores are organized into morulares structures virtually without a wall (Figure 2b), until the expulsion of spores observed in different stages (Figures 2c and 2d). Majority of these steps can be observed in Figure 2c.

**Figure 3:**
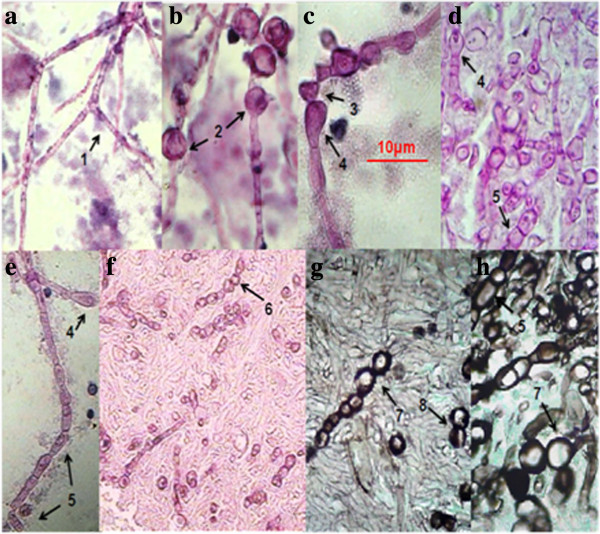
**Hyphae are forming pleomorphic cells. (a, b, c, d, f, g)** Septate and branched hyphae-1; arthroconidia production spherical-2, triangular-3, racket shaped-4, rectangular-5, barrel-shaped-6, ovoid-7, and an apparent state of cell budding-8. **(a, b, c, e)** Sputum and **(d, f, g, h)** lung tissue. 40X; **(a, b, c, d, e, f)** stained with Periodic acid –Schiff (PAS) and **(g, h)** the Grocott methenamine silver (GMS) staining protocol. Arrows indicate the structures described.

**Figure 4:**
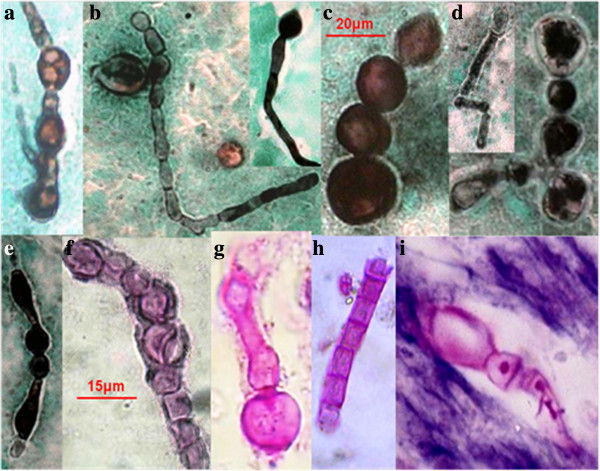
**Parasitic hyphal polymorphism of *Coccidioides*. (a, b, d)** Septate and branched hyphae; hyphae forming arthroconidia: spherical, ovoid, triangular, racket shaped, rectangular, and barrel-shaped. **(a, b, c, d, e, g, h)** Sputum, and **(i)** lung tissue. 40X. **(a, b, c, d, e)** staining with the Grocott methenamine silver (GMS) staining protocol, and **(f, g, h, i)** Periodic acid-Schiff (PAS).

**Figure 5:**
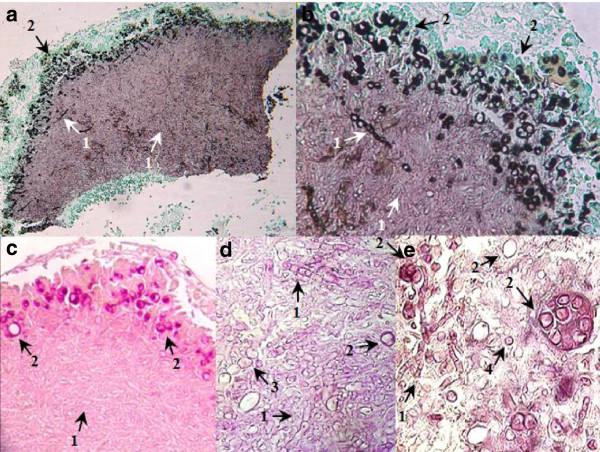
**Fungal ball formation.** Co-existence of hyphae and spherule, hyphae-1, spherules-2, hyphae-forming spherules-3, and endospores-4. Lung tissue. **(a)** 10X and **(b)** 40X Grocott methenamine silver (GMS) staining protocol. **(c)** 10X, **(e)** 40X Hematoxilin-eosin (H & E), and **(d)** 40X Periodic acid-Schiff (PAS). Arrows indicate the structures described.

**Figure 6:**
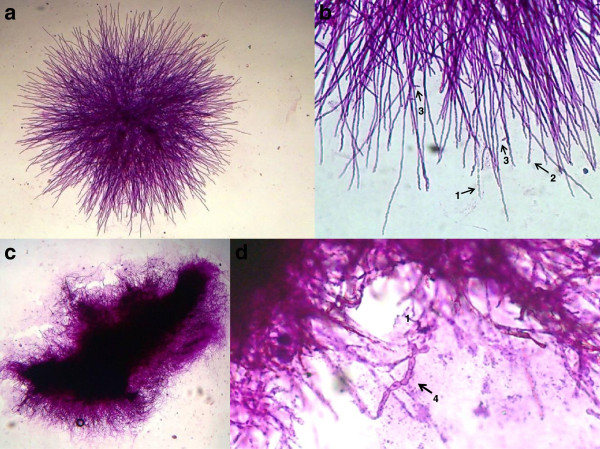
**Independent microcolonies of *Coccidioides *spp. (a)** Sea urchin-like microcolony; **(b)** filaments formed by septate hyphae with arthroconidia-1, hyphae sinuous-2 and hyphae branching-3 at 90°; **(c)** independent microcolony amorphous and furry; **(d)** hyphae forming barrel-shaped and racket-sharped arthroconidia-4. Bronchial lavage. **(a, c)** 10X and **(b, d)** 40X. **(a, b)** Hematoxilin-eosin (H&E), **(c, d)** Periodic acid-Schiff (PAS).

**Figure 7:**
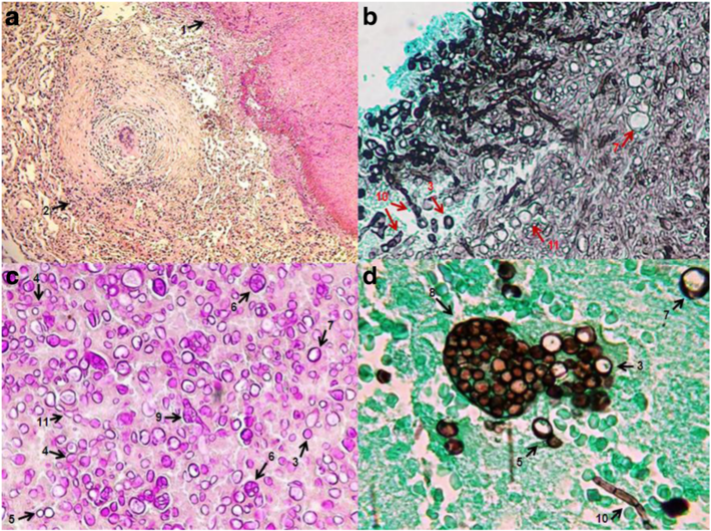
**Abridgement of the parasitic polymorphism of *Coccidioides *spp. (a)** microcolony with hifas/esferulas-1 coexisting with granuloma-2; **(b, c, d)** morphological differentiation: spores-3, spores producing hypha-4, spherules at different stages of maturation-5, with endospores-6, empty-7, releasing endospores-8 or producing hyphae-9; branching hyphae with barrel-sharped or racket-sharped-10, and ovoid/spherical-11 artroconidia. Lung tissue. **(a)** 10X, Periodic acid-Schiff (PAS); **(b)** 40X Grocott methenamine silver (GMS) staining protocol; **(c)** 40X, PAS **(d)** 100X GMS.

In Figure 3 the pleomorphism of mycelial forms is becoming notorious. We can found since typical mycelium narrow, with septum and lateral branches forming angles of 90 degrees (Figures 3a and 3e), until an atypical mycelium with forming diversified arthroconidia (Figures 3c, 3d, 3e, 3f, 3g, and 3h), as we have described previously. These mycelial forms may also cause confusion with other mycelial fungi that cause lung infection, mainly Hyfomicetes and may complicate the diagnostic laboratory work. The outstanding morphological diversity of hyphae is observed in all of the images of Figure 4; certain hyphae are generating spherical structures (arthroconidia) with spherule characteristics, such as a thick cell wall (Figures 4d and 4f), parasitic hyphal polymorphism, hyphae-forming ovoid and spherical cells, spherical, triangular, rectangular and barrel-sharped arthroconidia. Germination of spores and hyphal development was observed, initiating mycelium growth (Figures 4g and 4i). Finally, enterotalics hyphae are observed that are similar to the structures present in the of *Coccidioides* culture, however, here we present images of the fungus in its parasitic phase (Figure 4h). In Figure 5 Coexistence of spherules and hyphae in lung tissue is present in all of the patients studied. In the majority of specimens, we observed immature spherules, immature alone, or forming chains (Figures 5a, 5b, 5c and 5d). In a few patients, spherules with endospores were observed (Figure 5e). Microcolonies of *Coccidioides* are possibly confused with grains of pyogenic bacteria or with Actinomycetes grains (Figure 6). In Figure 7 we can observe a summary of all fungal structures noted in the parasitic phase of Coccidioides spp. In all of the specimens, in which the coexistence of hyphae and spherules were presented, each maturation step of Arthroconidia and endospores are also presented. Furthermore induction of granuloma as a mechanism of host response to the parasite was observed (Figure 7a). Some of these formations can be confused with other structures that are formed during other infections, such as tuberculosis and aspergillosis, blastomycosis, and Paracoccidioidomycosis, among others.

## Discussion

The diagnosis of coccidioidomycosis is complicated; this fungus is described as “The Great Simulator”, due to that it shares histopathologic clinical, radiological, and cytological characteristics with other fungal, bacterial, and/or parasitic infections. In order to make an accurate diagnosis of coccidioidomycosis, we recommended an integrated diagnosis that includes the following: epidemiological, clinical, and radiological data; microbiological studies (including isolation of the fungus); fresh examinations, cytological, histopathological and immunological studies. Microscopic laboratory diagnosis of Coccidioidomycosis includes analysis by observing the specimens in their fresh examination state with 15% KOH, cytology, or histopathological biopsies. This analysis is fast and inexpensive. Typical parasitic forms of *Coccidioides* comprise spherules and spherules/endospores; these provided an accurate diagnosis of coccidioidal infection. In contrast to this classical description of the *Coccidioides* spp. parasitic phase, our working group found a parasitic polymorphism in specimens from patients with a clinical and microbiological diagnosis of pulmonary coccidioidomycosis. Whith respect to the clinical forms of coccidioidomycosis and the presence of parasitic structures of *Coccidioides* spp., our group has found, in specimens, from patients with acute pulmonary coccidioidomycosis (evolution time, about 0.5 years), spherules with or without endospores uniquely, while in samples from patients with cavity chronic pulmonary infection (from 8 months to 2.5 years), as well as in patients with these characteristics and associated type 2 diabetes mellitus, we observed the presence of hyphae co-existing with spherules and a wide range of *Coccidioides* spp. parasitic fungal structures [7,8]. In specimens from patients with cavity chronic pulmonary coccidioidomycosis arthroconidia-to-spherule differentiation has been found (widely described), while others, such as spherule or endospore filamentation, hyphal polymorphism, and hyphae fragmentation into polymorphic arthroconidia have been described by only a few researchers in specimens from patients with chronic and cavitary pulmonary, ventriculoperitoneal shunt, and Central nervous system (CNS) *Coccidioides* spp. infection [1-6,9]. Although parasitic mycelial forms of *Coccidioides* spp. have been found in ≥50% of patients with chronic cavitary coccidioidomycosis, these are not diagnosed by themselves, when only cytological and histopathological methods are used; thus, it is advisable to research spherules and support this diagnosis by fungus culture or immunological test [7,8,11].

We present images of the parasitic polymorphisms of *Coccidioides* spp. observed in specimens from patients with cavitary chronic pulmonary coccidioidomycosis and their possible maturation and differentiation from these fungal structures during the parasitic cycle. Dimorphism begins with the isotropic growth of arthroconidia by spherical-cell enlargement and cell rounding and swelling, followed by synchronous nuclear division and segmentation. The central portion of the young spherule is occupied by a vacuole. Progressive compartmentalization of the cytoplasm surrounding the vacuole gives rise to uninucleate compartments that reproduce by mitosis and that differentiate into endospores. These processes can be observed in Figures 1 and 2. The mature spherule is 30–80 μm in diameter and may contain 200–400, and up to 800 endospores, spherule breaks, and endospores release (Figure 1 and Figure 2). The endospores can follow two pathways: a) producing mycelial growth forming hyphal polymorphism (Figure 3), these hyphae fragmented into arthroconia with pleomorphic shapes (spherical, triangular, barrel-shaped, ovoid, etc.) (Figure 3 and Figure 4), which can differentiate into spherule, or b) producing endospores that can differentiate directly into spherules. Thus, at any given point of parasitic cycle, the infected host is exposed to immature, mature, and rupturing spherules, newly released endospores, arthroconidia, and mycelial forms (Figures 1, 2, 3, 4, 5, 6, 7). The integration of parasitic mycelial forms of *Coccidioides* spp. was already published in 2008 by the same authors. In the present article, the large number of parasitic structures observed is presented and the concept of the parasitic polymorphism of *Coccidioides*spp. was supported by the diversity of fungal structures is presented [8,9,11-13] and this report.

We thing that the development of the parasitic polymorphism of *Coccidioides* spp. is the outcome of host-parasite interaction, in which the immune response and fungal virulence factors are decisive for the presence of multiple fungal structures (described in more detail by Muñoz-Hernandez et al. [11]). *Quorum sensing*, or perception of *quorum*, is defined as the microorganisms’ mechanism for intercellular communication that controls gene expression in terms of cell density. *Quorum sensing* involves multiple mechanisms, such as Histidine kinases (HK), exopolysaccharide production (biofilm production), dimorphism, sporulation, production of secondary metabolites, induction of virulence factors, etc. [14,15]. Morphogenesis of the fungus is regulated by *quorum sensing* processes, HK regulation, and N-acetyl glucosamine (GlcNAc) response, among others [14-18]. Each of these processes responds to the sensory stimuli that surround the fungus, including CO_2_ concentration, pH, chronic infection, the genetic predisposition of the racial type host, parasitized host tissue (lung, CNS, ventriculoperitoneal shunt and cavities), and the host’s immunologic status, as well as others). The parasitic polymorphism described in this study supports this adaptation of the fungus because in its parasitic phase, it can be expressed in diverse fungal structures (mycelia, arthroconidia, endospores, and spherules) in response to its adaptation as a parasite. In chronic infection, the pathogen can establish the conditions of nutrition (arginase activity generated an amino acid pool), alkaline pH (urease activity), O_2_ concentration, tissue damage, and evasion of the immune response with morphological changes, which ultimately lead to prolonged survival of the fungus and its successful adaptation to parasitism [17-29].

The parasitic polymorphism of *Coccidioides* spp. described in this study could be explained if each fungal cell receives the specific stimuli that closely surround it, and if each cell has the ability to respond to these stimuli, possibly with morphological changes related with a sensing factor response. Indeed, response to sensed factors would favor the dominance of certain fungal structures over others, or the co-existence of several structures at the same site (Figure 5 and Figure 7). The parasitic polymorphism of *Coccidioides* spp. involves diverse morphologies generated by multiple factors such as following: virulence factor; clinical form; co-morbidity; the human race, and humoral and cellular immune response.

Furthermore, our research group suggests the integrating by means of the recently published bibliographic information, in which the presence of both typical parasitic forms (spherules and spherules/endospores) as well as atypical parasitic forms (mycelium, hyphae and polymorphic arthroconidia) could be associated with a predominant response adaptation to the parasitic state of *Coccidioides* spp. due to comparative transcriptomics of its saprobic and parasitic phases. Gene analyses showed that this fungus has lost genes associated with plant cell-wall-degrading enzymes such as cellulases, cutinases, tannases or pectinesterases, and with genes involved in its sugar metabolism; In addition, this fungus has gene duplication associated with parasitism, such as deuterolysin (M35) metalloproteases (*Mep2-*like to *Mep8*-like). Nevertheless, this point of view comprises only one possible explanation for the parasite polymorphism of *Coccidioides* spp. and we do not attempt in any way to prove it [30,31].

*Coccidioides* spp. parasitic polymorphism is poorly known by Microbiologists in terms of diagnosis of coccidioidomycosis through observation of the specimens, in their fresh examination state with KOH, cytology or histopathological biopsies; hyphae and arthroconidia of *Coccidioides* are not diagnosed by themselves, but when only cytological and histopatological methods for diagnosis are used. Indeed, it is important to diffuse this knowledge of the diverse forms generated by this fungus in its parasitic cycle in order to Microbiologists to be able to make an accurate diagnosis, to know the pathophysiology of the infection, and to propose alternatives with therapeutic aims for supporting the cure and improvement of the patient.

It is important to observe the spreading of the parasite polymorphism of *Coccidioides* in order to perform early and accurate diagnosis of this disease; it is probable that the microhabitat, generated within the host for the presence of the fungus, allows this polymorphic parasitic expression. In conclusion, arthroconidia inhaled by a susceptible host engage in a phenotypic morphological transition from the mycelial to the parasitic phase presenting diverse morphologies, and according to the diversity of parasitic forms observed in specimens from patients with coccidioidomycosis, it is possible to consider *Coccidioides* as a polymorphic fungus in its parasitic phase.

## Conclusions

•According to the diversity of parasitic forms observed in specimens from patient with coccidioidomycosis, it is possible to consider *Coccidioides* as a polymorphic fungus in its parasitic phase.

•The arthroconidia inhaled by a susceptible host have phenotypic morphological transition from mycelial to the parasitic phase presenting diverse morphologies.

•Probably, the microhábitat, generated within the host for the presence of the fungus, allows this polymorphic parasitic expression.

•It is important spreading the parasite polymorphism of *Coccidioides* to perform early and accurate diagnosis of this disease.

## Abbreviations

HK: Histidine kinases; GlcNAc: N-acetyl glucosamine response; H&-E: Hematoxylin and eosin; GMS: Gomori methenamine silver; PAS: Periodic acid Schiff; CFW: Calcofluor white; SOW: Spherule outer wall; SWOgp: SOW is coated with glycoprotein; MEP1: metalloproteinase gene; UV: Ultraviolet; iNOS: inducible Nitric oxide synthase; NO: Nitric oxide.

## Competing interests

All authors have declared that there do not have commercial or other associations that might pose a conflict of interest.

## Authors’ contributions

**BM** Participated in the study design, study of images from specimens of smears with 15% potassium hydroxide, cytology, and tissue biopsies of a histopathologic collection from patients, and helped to draft the manuscript. **GP** Carried out the serologic tests, participated in the clinic studies and patient data case studies and sensitivity test for cellular immune response. **CC** Participated in the study design and assistance on administrative procedures. **MAM** Participated in the study design, coordination and draft the manuscript. All authors read and approved the final manuscript.

## Pre-publication history

The pre-publication history for this paper can be accessed here:

http://www.biomedcentral.com/1471-2334/14/213/prepub
